# Regadenoson in Europe: first-year experience of regadenoson stress combined with submaximal exercise in patients undergoing myocardial perfusion scintigraphy

**DOI:** 10.1007/s00259-013-2619-0

**Published:** 2013-11-22

**Authors:** M. Brinkert, E. Reyes, S. Walker, K. Latus, A. Maenhout, R. Mizumoto, C. Nkomo, K. Standbridge, K. Wechalekar, S. R. Underwood

**Affiliations:** 1 Royal Brompton Hospital, Sydney St, London, SW3 6NP UK; 2 National Heart & Lung Institute, Imperial College London, London, UK

**Keywords:** Myocardial perfusion scintigraphy, Regadenoson, Side effects, Safety, Tolerability

## Abstract

**Purpose:**

Regadenoson was approved for clinical use in Europe in 2011. Since then, it has become the default form of stress at our institution. We have assessed the side-effect profile and tolerability of regadenoson in patients undergoing clinically indicated myocardial perfusion scintigraphy between July 2011 and July 2012.

**Methods:**

Clinical, stress and imaging data were recorded prospectively. Symptoms during stress were recorded and defined as mild, moderate or severe. An adverse event was defined as any symptom that persisted for more than 30 min or that required investigation or treatment.

**Results:**

Of 1,764 consecutive patients, 1,581 (90 %) received regadenoson combined with submaximal exercise unless contraindicated. Symptoms were common (63 %) but transient and well-tolerated. The severity of symptoms was recorded in most patients as mild (84 %). Dyspnoea (36 %) and chest discomfort (12 %) were the commonest side effects. Adverse events were reported in eight patients (0.5 %), thought to be vasovagal in seven of these. All patients recovered fully without sequelae. There were no deaths, myocardial infarction or hospital admissions. Regadenoson stress was performed in 206 patients (12 %) with asthma or chronic obstructive pulmonary disease (COPD) without bronchospasm or any other major side effect.

**Conclusion:**

We studied the symptom profile of regadenoson in the largest European cohort to date. Regadenoson combined with submaximal exercise was well tolerated, notably also in patients with asthma or COPD. The majority of regadenoson-related adverse events were vasovagal episodes without sequelae.

## Introduction

Although dynamic exercise is the preferred form of stress for myocardial perfusion scintigraphy (MPS), many patients are unable to exercise maximally and pharmacological stress is common [1]. At our institution, pharmacological stress is the default form of stress because most patients have already been considered for or have undergone an exercise ECG. An efficient and effective way of stressing coronary function in this setting is with the use of vasodilators. Adenosine and dipyridamole are both potent primary coronary vasodilators with a nonselective action, which explains the side effects commonly associated with these agents [2–9]. To avoid side effects and potential adverse events, a number of selective adenosine A_2A_ receptor agonists have been developed [10–14]. Of these, regadenoson is the only one approved for clinical use by the Food and Drug Administration. Approval was granted by the European Medicines Agency in 2010 and by the Royal Brompton & Harefield NHS Foundation Trust New Drugs and Clinical Guidelines Committee in May 2011. Since July 2011, we have used regadenoson as the default form of stress, and we now have the largest experience outside North America. The main objective of this study was to assess the side effect profile, safety and tolerability of regadenoson in patients undergoing clinically indicated MPS. A secondary objective of the study was to compare this experience with other forms of cardiac stress.

## Methods

### Patient selection

**Fig. 1:**
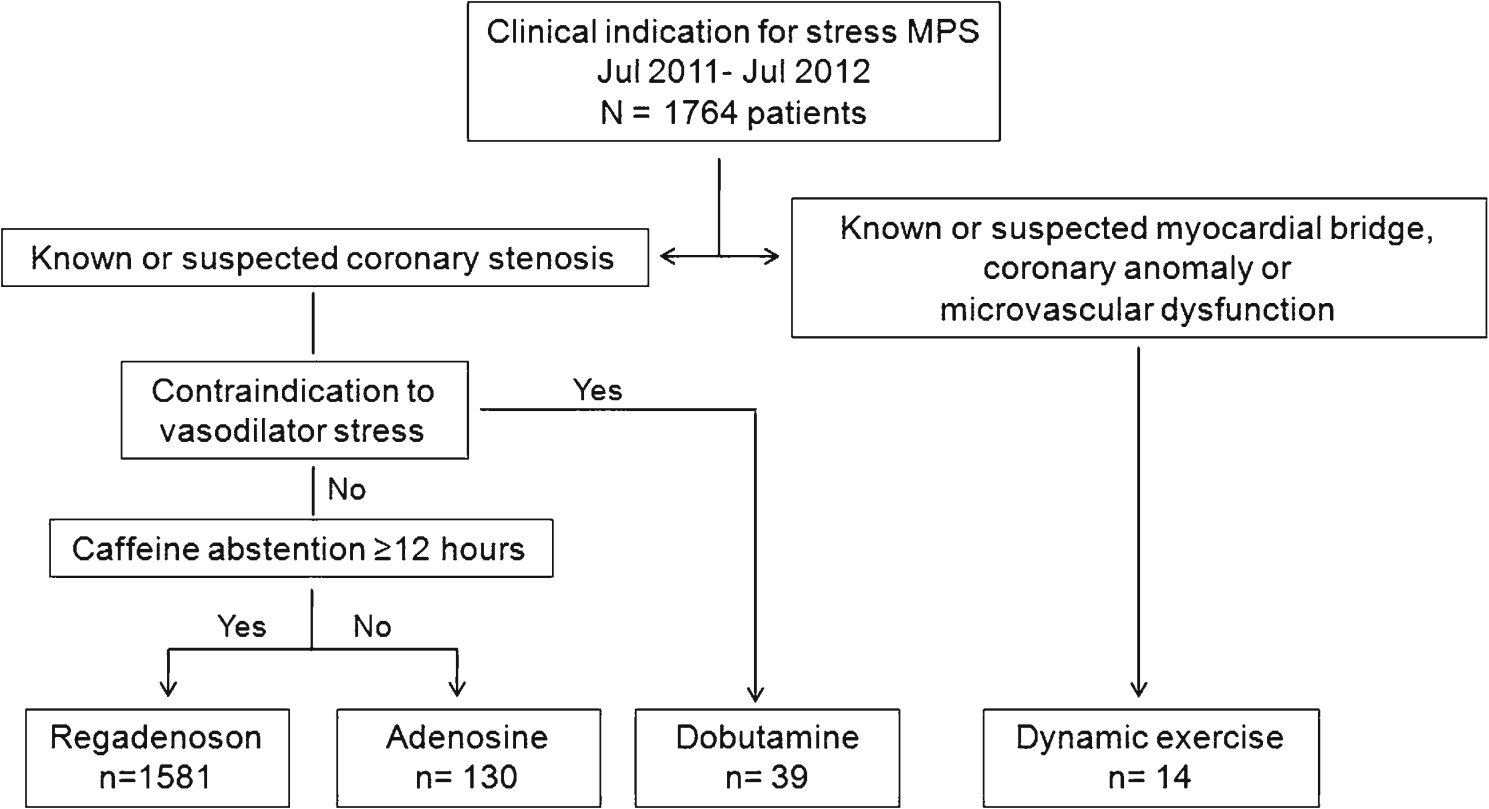
Flow diagram showing patient selection for stress testing according to routine clinical practice

Regadenoson stress was implemented at our institution on 22 July 2011. As part of an audit of clinical practice, we studied all patients who underwent clinically indicated MPS over the following 12 months. Clinical and imaging data were collected prospectively and retrieved for retrospective analysis from a dedicated database. The selection of stress agent was a clinical decision based upon using regadenoson by default with patients receiving another form of stress if this was preferred over regadenoson or there was a contraindication to the use of this agent (Fig. 1). Hence, in patients with suspected myocardial bridging, coronary anomaly or microvascular dysfunction as the primary cause of symptoms, dynamic exercise was the preferred form of stress. Patients who were suitable for vasodilator stress but self-reported caffeine consumption within 12 h of the test were given high-dose adenosine (210 μg/kg/min) to overcome the antagonism mediated by caffeine on the adenosine receptors [15]. Patients with a contraindication to vasodilator stress received dobutamine. After 3 months, regadenoson was given to patients with obstructive airways disease irrespective of disease severity.

### Symptoms and adverse events

Symptoms during stress were recorded and defined as mild, moderate or severe by the health professional (a clinical nurse specialist or a nuclear cardiologist) conducting the stress test. A mild symptom did not distress the patient, a moderate symptom did distress the patient but was self-limiting without the need for intervention, and a severe symptom distressed the patient and required some form of intervention to alleviate it. An adverse event was defined as any symptom that persisted for more than 30 min or that required investigation or treatment, for instance reversal of the stress agent by aminophylline.

### Stress MPS

Regadenoson was administered intravenously as a rapid injection of 400 μg over at least 10 s followed by a 5-ml saline flush. The radiopharmaceutical was administered intravenously approximately 20 s after regadenoson injection. Unless there was left bundle branch block or paced rhythm, regadenoson was combined with bicycle exercise at 0 W (free wheel), or 5, 10, 15 or 25 W depending on exercise tolerance. Supplemental exercise was initiated 1 min before regadenoson injection and continued for 2 – 3 min after injection of regadenoson. ECG rhythm was monitored throughout and heart rate and blood pressure recorded every 2 min or more often if necessary until completion of the test. Patients with airways disease had regadenoson without pretreatment with a bronchodilator. To antagonize the effect of caffeine in patients who self-reported caffeine ingestion within 12 h before the test, adenosine was infused at a high dose of 210 μg/kg/min over 6 min with supplemental exercise. Dobutamine was given intravenously at incremental doses of 5 – 10 μg/kg/min every 3 – 5 min starting at 5 – 10 μg/kg/min up to a maximal dose of 40 μg/kg/min. Dynamic exercise was performed on a bicycle ergometer at increasing workloads of 25 – 50 W every 2 – 3 min until ≥85 % of maximum predicted heart rate was reached or angina and ischaemic ECG changes developed.

### Imaging analysis

All images were interpreted according to routine clinical practice by an experienced nuclear cardiologist. For the purpose of this study, further analysis was conducted in patients who experienced an adverse event following regadenoson injection. Quantitative perfusion SPECT software (QPS, version 2008; Cedars-Sinai Medical Center, Los Angeles, CA) was used to calculate the total burden of ischaemia as the total perfusion deficit (TPD) difference between stress and rest images. Total ischaemic burden was defined as mild, moderate or severe if TPD was <6 %, 6 – 9 % or ≥10 %, respectively. In addition, the stress and rest summed segmental scores were calculated using a 17-segment model with each segment scored from 0 to 4 (0 normal, 1 mild, 2 moderate, 3 severe defect and 4 absent tracer uptake). The extent and severity of ischaemia was defined as the difference between summed stress and summed rest scores (SDS). A SDS <6 was considered mild, 7 – 12 moderate and >12 severe [16]. Left ventricular ejection fraction was computed using Quantitative Gated SPECT software (QGS, version 2008; Cedars-Sinai Medical Center).

### Statistical analysis

Data summary and statistical comparisons were performed using Microsoft Excel Analysis Toolpak and SPSS (IBM SPSS Statistics v20). Continuous variables were compared using analysis of variance; categorical variables were compared using the chi-squared test. Logistic regression analysis was performed to examine the association between stress-induced adverse events and potential predictors of such events.

## Results

### Patient characteristics

A total of 1,764 consecutive patients underwent stress MPS between 22 July 2011 and 28 July 2012. Of these, 69 % were male; mean ± SD age was 67 ± 11 years and mean ± SD body mass index 28 ± 5 kg/m^2^. The majority of patients (90 %) underwent regadenoson stress while the remainder had adenosine (7 %), dobutamine (2 %) or dynamic exercise (1 %). The prevalence of cardiovascular risk factors and other characteristics of the population are listed in Table 1. There were no significant differences between groups except for lung disease, which was more common (83 %) in the dobutamine group. Symptoms on presentation are shown in Table 2. Of the 1,764 patients, 73 % had a history of chest pain, and in 24 % of these patients the chest pain was angina, 60 % had dyspnoea and 9 % had a history of heart failure, but less than 1 % had severe heart failure. Dyspnoea was more frequent in the dobutamine group, and there was a small excess of patients with a history of heart failure in the adenosine and dobutamine groups.

**Table 1:** Patient demographics and clinical characteristics

	Global		Adenosine		Regadenoson		Dobutamine		Exercise		*P*
	*n*	%	*n*	%	*n*	%	*n*	%	*n*	%	
No. of patients											
Total	1,764	100	130	7	1,581	90	39	2	14	1	n/a
Male	1,211	69	96	74	1,079	68	26	67	10	71	0.6
Age (years), mean ± SD	67 ± 11	–	69 ± 12	–	67 ± 11	–	68 ± 13	–	56 ± 25	–	<0.001
BMI (kg/m^2^), mean ± SD	28 ± 5	–	27 ± 5	–	28 ± 5	–	30 ± 8	–	27 ± 4	–	0.01
Diabetes	411	24	29	23	369	23	12	31	1	7	0.3
Hypertension	1,300	74	96	74	1,167	74	27	69	10	71	0,9
Hyperlipidaemia	1324	76	102	78	1,189	75	24	62	9	64	0.3
Smokers	135	11	16	19	116	7	3	8	0	0	0.1
Known coronary artery disease	1023	59	80	63	921	58	17	44	5	36	0.1
Myocardial infarction	460	26	37	28	413	26	7	18	3	21	0.7
Percutaneous coronary intervention	627	36	43	33	571	36	10	26	3	21	0.4
Coronary artery bypass grafting	285	16	28	22	251	16	4	10	2	14	0.3
Lung disease	304	18	25	20	246	16	30	83	3	25	<0.001

*P* values are between stress groups (statistical significance at *P* < 0.05)

**Table 2:** Clinical history and presenting symptoms

	Global		Adenosine		Regadenoson		Dobutamine		Exercise		*P*
	*n*	%	*n*	%	*n*	%	*n*	%	*n*	%	
Chest discomfort											
None	475	27	29	22	431	27	9	23	5	36	>0.5
Non-anginal	8	0	0	0	8	1	0	0	0	0	
Atypical chest pain	641	36	46	35	572	36	15	38	6	43	
Angina	430	24	34	26	388	25	10	26	1	7	
CCS 1	274	66	22	69	244	65	7	64	1	100	
CCS 2	99	24	4	13	91	24	4	36	0	0	
CCS 3	42	10	6	19	39	10	0	0	0	0	
CCS 4	0	0	0	0	0	0	0	0	0	0	
Nonspecified chest pain	209	12	21	16	181	11	5	13	2	14	
Heart failure	137	9	20	17	110	8	6	21	1	8	0.002
NYHA 1	51	3	5	4	43	3	2	7	1	8	
NYHA 2	40	3	5	4	32	2	3	11	0	0	
NYHA 3	41	3	10	8	31	2	0	0	0	0	
NYHA 4	5	0	0	0	4	0	1	4	0	0	
Dyspnoea	1,041	60	87	70	915	59	33	89	6	43	0.001
Mild	637	37	43	34	583	37	9	24	2	14	
Moderate	332	19	35	28	273	18	21	57	3	21	
Severe	72	4	9	7	59	4	3	8	1	7	

*CCS* Canadian Cardiovascular Society angina class, *NYHA* New York Heart Association heart failure class

Statistical significance at *P* < 0.05

### Haemodynamic response to stress

**Fig. 2:**
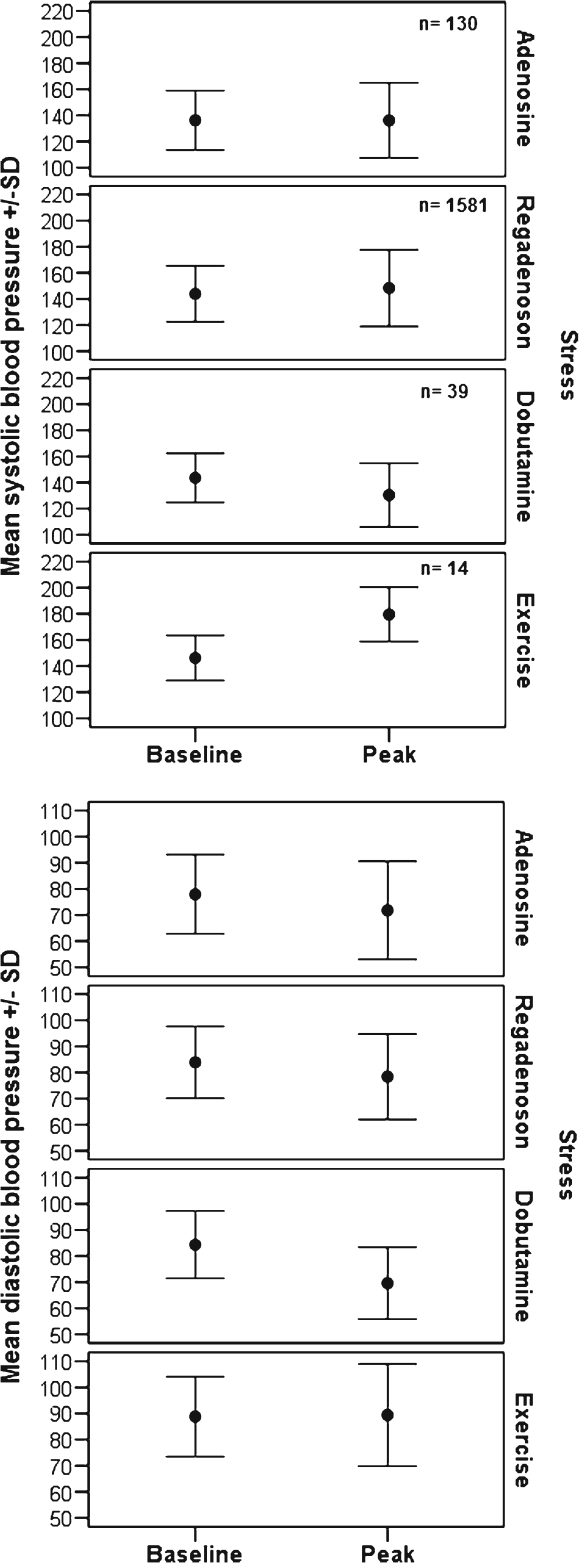
Blood pressure response to stress. **a** Baseline and peak systolic blood pressure; **b** baseline and peak diastolic blood pressure. Baseline measures were obtained immediately before starting the stress test. Peak blood pressure was that with the largest change over the duration of the test. Values are means ± 1 standard deviation

**Fig. 3:**
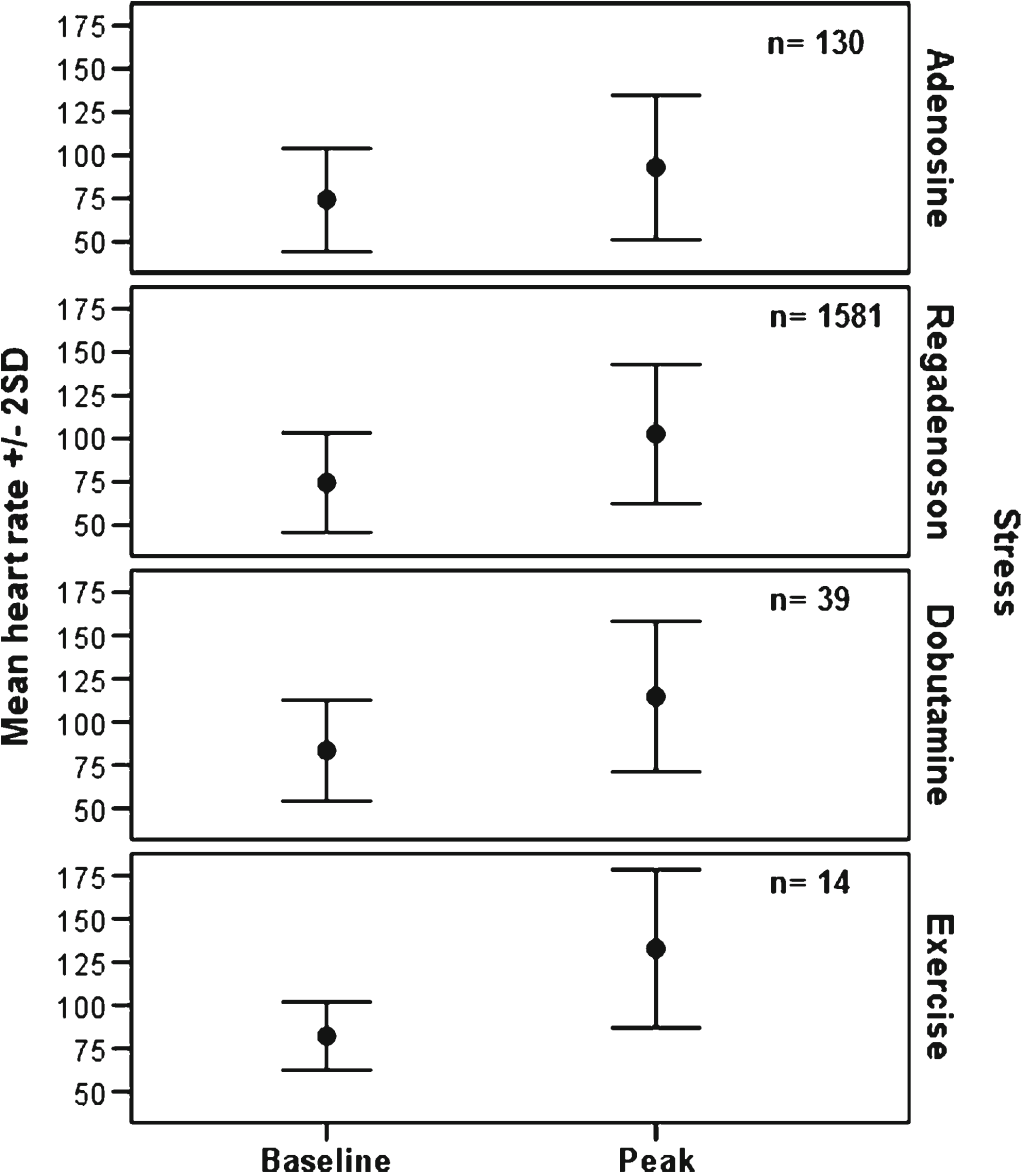
Heart rate at baseline and peak stress. Baseline measures were obtained immediately before starting the stress test. Peak heart rate was that with the largest change over the duration of the test. Values are means ± 2 standard deviation

There was a statistically significant but clinically unimportant difference in resting blood pressure between groups (Fig. 2). Heart rate increased by 28 ± 17 bpm with regadenoson stress, 19 ± 18 bpm with adenosine, 31 ± 24 bpm with dobutamine and 51 ± 24 bpm with dynamic exercise (Fig. 3). There was no significant change in blood pressure from rest to peak stress in the regadenoson and adenosine groups, while there was a significant reduction in the dobutamine group. The haemodynamic response to vasodilators was modified by concurrent dynamic exercise: median workload 25 W (0 W, 100 W) for regadenoson vs. 15 W (0 W, 75 W) for adenosine.

### Symptoms and side effects

**Fig. 4:**
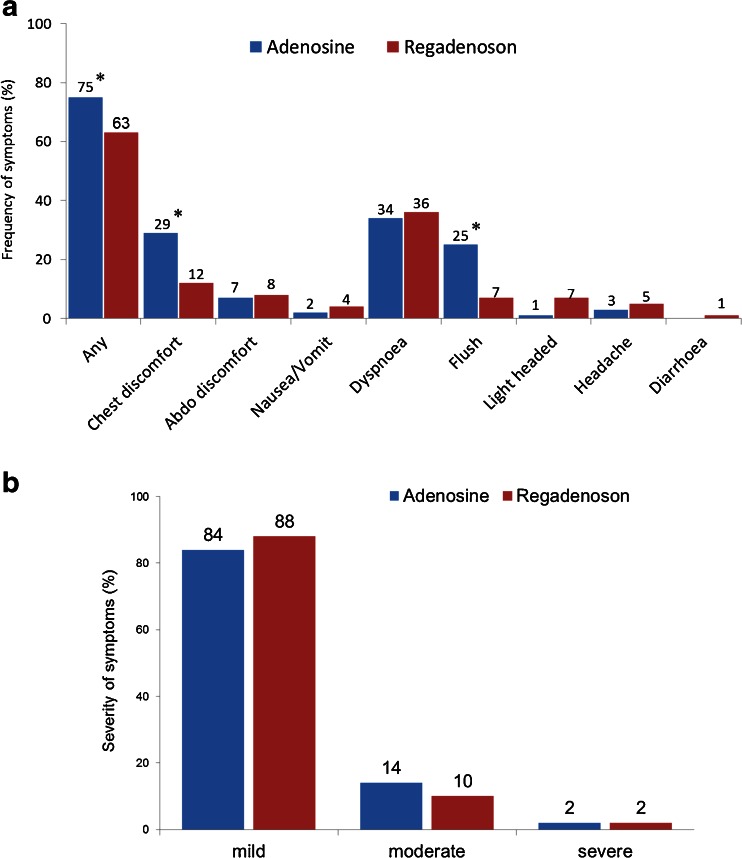
Symptoms during stress: **a** frequency; **b** severity. *P ≤ 0.05

Symptoms were common with all forms of pharmacological stress with 63 % of patients in the regadenoson group, 75 % of patients in the adenosine group, 46 % in the dobutamine group and 36 % in the exercise group (*P* = 0.001, Fig. 4a) reporting at least one symptom. Dyspnoea (36 %) and chest discomfort (12 %) were the commonest side effects following regadenoson injection. Chest discomfort and flushing occurred more often with adenosine (29 % and 25 %, respectively) than with regadenoson (12 % and 7 %, respectively), but light-headedness was more common with regadenoson (7 % vs. 1 % for adenosine; Table 3). Diarrhoea was reported by 12 patients (1 %) in the regadenoson group compared with none in the other groups, but this difference was not statistically significance (*P* = 0.9). Chest discomfort and dyspnoea were the commonest side effects in the dobutamine group while dyspnoea was the most frequent side effect in the exercise group. The severity of symptoms did not differ significantly between stress agents (*P* = 0.3) with most symptoms graded as mild: 84 %, 88 %, 82 % and 60 % in the regadenoson, adenosine, dobutamine and exercise groups, respectively (Table 4, Fig. 4b).

**Table 3:** Symptoms and adverse events

Event	Adenosine		Regadenoson		Dobutamine		Dynamic		*P*
	*n*	%	*n*	%	*n*	%	*n*	%	
Any	97	75	996	63	18	46	5	36	0.001
Chest discomfort	38	29	193	12	11	28	1	7	<0.001
Palpitations	0	0	11	1	1	3	0	0	0.2
Abdominal discomfort	9	7	132	8	0	0	0	0	0.2
Nausea/vomiting	2	2	56	4	1	3	0	0	0.7
Diarrhoea	0	0	12	1	0	0	0	0	0.9
Dyspnoea	44	34	565	36	7	18	4	29	0.1
Flushing	32	25	114	7	0	0	0	0	<0.001
Light-headedness	1	1	114	7	2	5	0	0	0.1
Headache	4	3	86	5	1	3	0	0	0.8
Fatigue	4	3	17	1	0	0	0	0	0.5

Statistical significance at *P* < 0.05

**Table 4:** Severity of symptoms

Severity	Adenosine		Regadenoson		Dobutamine		Dynamic		*P*
	*n*	%	*n*	%	*n*	%	*n*	%	
Mild	90	84	939	88	14	82	3	60	0.3
Moderate	15	14	108	10	3	18	2	40	
Severe	2	2	18	2	0	0	0	0	

Statistical significance at *P* < 0.05

### Adverse events

There were no deaths, myocardial infarction or hospital admission following stress MPS. Adverse events were observed in eight patients (0.5 %), all in the regadenoson group (Table 5). Seven of the eight events were episodes of symptomatic hypotension with inappropriate bradycardia or failure of compensatory tachycardia, and they were therefore classified as vasovagal. One of these occurred after intravenous cannulation and before regadenoson injection, and the others occurred between 2 and 10 min after injection. Two episodes occurred in patients who did not exercise. Of all vasovagal events, three were moderate and two severe; the two severe episodes progressed to sinus arrest and asystole lasting 30 s and 10 s and requiring CPR. Aminophylline was administered to three patients. All patients recovered fully without sequelae. No association was found between the incidence of adverse events during stress and clinical and imaging characteristics including known coronary artery disease (CAD), older age (>65 years), cardiovascular risk factors and inducible ischaemia on MPS (*P* > 0.05 for all). In the regadenoson group, there was a significant reduction in heart rate and systolic blood pressure during stress in patients who experienced at least one adverse event compared with those who remained free of complications (heart rate −6 ± 18 bpm vs. 28 ± 16 bpm, *P* = 0.001; and systolic blood pressure −36 ± 34 mmHg vs. 5 ± 26 mmHg, *P* = 0.01). Only heart rate and systolic blood pressure reduction during stress predicted the likelihood of an adverse event after regadenoson injection (*P* > 0.01 for both).

**Table 5:** Adverse events and imaging findings in patients with at least one adverse event during regadenoson stress testing

Patient demographics		Clinical findings			Adverse events				MPS results				
Age (years)	Sex	BMI (kg/m^2^)	Ethnicity	History	Event	Severity	Description	Management	Tracer	Summed difference score	Ischaemic total perfusion deficit	Ischaemia location	Left ventricular ejection fraction at rest (%)
53	M	23	Caucasian	None	Vasovagal	Moderate	Pulse 70, blood pressure 85/40	Self-resolved	^99m^Tc-tetrofosmin	0	0	No ischaemia	66
54	F	24	Arabic	Atypical chest pain	Vasovagal	Moderate	Pulse 72, blood pressure 75/40	Aminophylline	^201^Tl	3	3	Inferolateral wall	82
58	M	36	Caucasian	Myocardial infarction, percutaneous coronary intervention, stable angina	Vasovagal	Moderate	Pulse 50, blood pressure 70/40	Self-resolved	^99m^Tc-tetrofosmin	3	3	Inferior wall	52
67	M	30	Caucasian	Coronary artery bypass grafting	Vasovagal	Mild	Pulse 56, blood pressure 85/70 after cannulation and before stress injection		^201^Tl	10	8	Anteroapical and inferolateral walls	61
67	M	33	Asian	Myocardial infarction, prior syncope after intravenous cannulation	Vasovagal	Severe	10-s asystole	CPR	^99m^Tc-tetrofosmin	3	5	Anteroseptal wall	44
75	F	27	Caucasian	mild stable angina	Vasovagal	Moderate	Pulse 58, blood pressure 70/55	Aminophylline	^201^Tl	0	0	No ischaemia	79
75	F	33	Asian	Atypical chest pain	Vasovagal	Severe	30-s asystole	Aminophylline, CPR	No scan				
79	M	24	Arabic	Myocardial infarction, no lung disease	Bronchospasm	Mild		Resolved rapidly after salbutamol	^201^Tl	4	3	Anterolateral wall	59

### Lung disease

Lung disease including airways obstruction was documented by clinical history in 297 patients (17 %) of whom 145 (49 %) had asthma, 111 (37 %) chronic obstructive pulmonary disease (COPD), 34 (11 %) interstitial lung disease and 7 (2 %) bronchiectasis. Of these 297 patients, 242 (81 %) received regadenoson. Of all patients with a history of asthma in the regadenoson group, 71 % had intermittent symptoms, 26 % mild persistent symptoms, and 3 % moderate symptoms, and none had severe persistent asthma. No patient developed bronchospasm with regadenoson, but one patient (male, 79 years old) with a history of previous myocardial infarction (MI) but not known to have airways disease developed mild bronchospasm. The bronchospasm developed soon after injection of regadenoson and resolved shortly after inhalation of salbutamol. Most patients in the dobutamine group (77 %) had some form of lung disease, mainly asthma or COPD. Of these, 13 % had intermittent asthma, 19 % mild persistent asthma, 44 % moderate persistent asthma and 15 % severe persistent asthma. None of the patients who underwent dobutamine stress experienced any respiratory complications.

### Imaging results

**Fig. 5:**
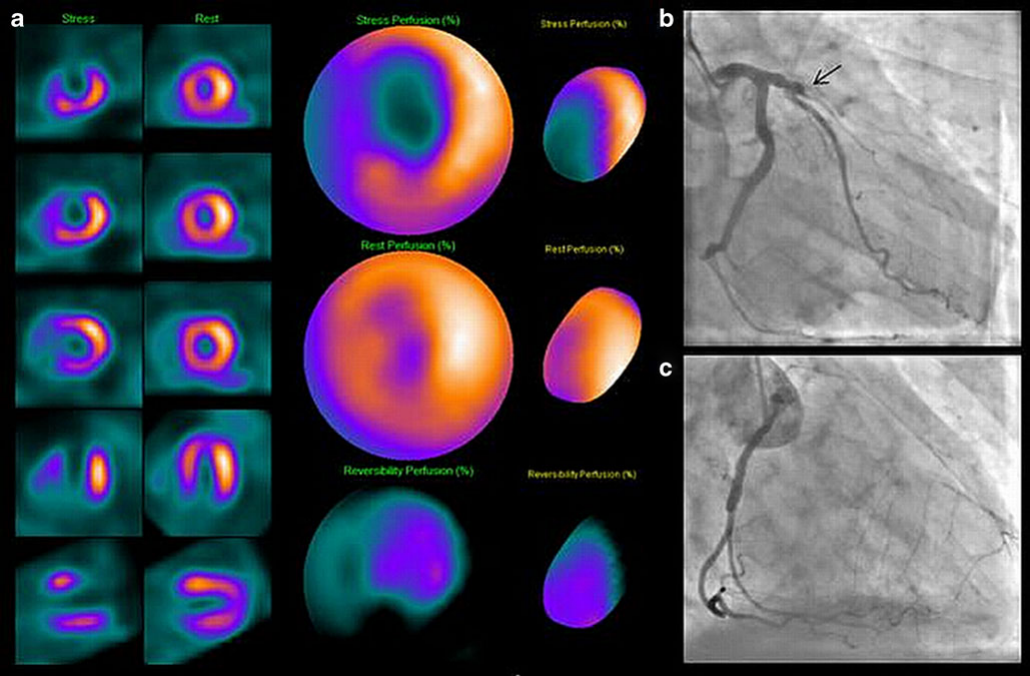
A 71-year-old man with chest pain of suspected cardiac origin. **a** Regadenoson/rest ^201^Tl MPS. The left ventricular tomograms and the corresponding polar maps show an extensive inducible perfusion abnormality in the left anterior descending coronary artery (LAD) territory. **b**, **c** X-ray coronary angiography demonstrates (**b**) total LAD occlusion (*arrow*) and (**c**) collateralization from the right coronary artery

A total of 673 MPS studies (38 %) were normal. Among abnormal scans, a reversible or inducible myocardial perfusion abnormality was present in 946 (54 %). A representative patient is shown in Fig. 5. Of these, 366 (21 %) studies showed an additional fixed abnormality consistent with myocardial damage (full- or partial-thickness infarction). No inducible ischaemia but evidence of infarction was observed in the remaining 141 patients (8 %). Four studies were considered non-diagnostic (0.2 %). Among patients who experienced at least one stress-related adverse event (*n* = 8), mild to moderate inducible ischaemia was present in 5 of 7 MPS studies; none of the studies showed severe ischaemia or severe left ventricular dysfunction on ECG-gated images (Table 5). In one patient with severe vasovagal response, imaging was delayed >45 min post-thallium injection and hence no stress images were obtained.

## Discussion

This report summarizes our first year of experience performing regadenoson stress in patients with suspected or known CAD. To the best of our knowledge, this is the largest series of patients undergoing clinically indicated regadenoson MPS in Europe. These results confirm previous observations and show that regadenoson in this setting is well tolerated and safe with no deaths, hard cardiac events or hospital admission following its administration. The rate of other untoward events was 0.5 % and these were mainly vasovagal episodes that resolved without sequelae, although two patients received CPR as part of medical management.

Our results are very similar to those of previous studies including the phase III ADVANCE clinical trials. In these studies, side effects were reported in slightly over 70 % of patients undergoing regadenoson stress [17, 18]. In the current study, just over 60 % of patients experienced at least one side effect following regadenoson injection. The lower frequency of side effects in the current study might be explained by the addition of submaximal exercise, which is known to reduce the frequency and severity of vasodilator-related side effects [19]. As in previous studies [17, 18], most side effects were mild and well tolerated. We have also previously shown that side effects are less common with increasing BMI, although this aspect was not assessed in the study [20].

As shown by previous comparisons between regadenoson and adenosine stress, the frequency and intensity of side effects after regadenoson injection is similar to that of adenosine, but the nature and quality of side effects differs between the two agents; for example, chest discomfort and flushing are more common with adenosine than regadenoson, while headache and gastrointestinal symptoms are more frequent with regadenoson [17, 18]. Differences in the degree of vasodilation and sympathetic stimulation as well as differences in affinity for the adenosine receptors may account for this finding [21].

Gastrointestinal symptoms and especially diarrhoea have the potential for causing procedural delays that may compromise image quality and accuracy, particularly when ^201^Tl is used. Nearly 1 % of our patients had diarrhoea following regadenoson injection; however, all episodes were self-limiting and images were obtained in all patients. The frequency of diarrhoea in our series was significantly lower than the 11 % found in a recent study [22]. It is possible that lack of awareness of causality between regadenoson and gastrointestinal symptoms and cultural differences might have prevented our patients from reporting this unpleasant side effect. However, it is also likely that the addition of dynamic exercise might have contributed to the lower frequency of gastrointestinal symptoms. Indeed, Thomas et al. have already shown that regadenoson combined with exercise is associated with a low frequency of gastrointestinal symptoms (5 %) [23].

Regadenoson was safe with no fatal or nonfatal cardiac events observed in the hours following its administration (up to 4 h after the procedure). There was no need for hospital admission following regadenoson stress and this is consistent with previous observations [17, 18]. No association was found between the incidence of adverse events and several clinical and imaging parameters. In contrast to previous reports, most untoward adverse events were episodes of symptomatic hypotension associated with absolute or relative bradycardia following regadenoson injection [17]. To the best of our knowledge no vasovagal episodes have been described before [18].

It is beyond the scope of this report to explain the mechanism of action by which regadenoson might cause vasovagal syncope but we can hypothesize. In general, some individuals are prone to experiencing vasovagal episodes secondary to any investigation [24–26]. In one patient, vasovagal symptoms and signs developed soon after cannulation and before regadenoson was given. All other episodes occurred after regadenoson injection. A direct bradycardic effect of regadenoson seems unlikely. Regadenoson has little if any effect on the sinoatrial and atrioventricular nodes as demonstrated in previous animal models [27]. As expected, the incidence of bradyarrhythmias including advanced degrees of atrioventricular block after regadenoson administration is low [22]. Experimental studies have demonstrated that regadenoson has no significant A_2B_ activity, and hence it should not cause peripheral vasodilation, although there may be some vasodilation mediated by peripheral A_2A_ receptors. This, however, would result in sympathetic activation with tachycardia through the baroreceptor reflex. Two of the patients with a vasovagal episode had abdominal discomfort, nausea or vomiting before hypotension and bradycardia. Susceptible patients may have indirect vagal stimulation in response to these symptoms after regadenoson injection [28, 29]. This effect could have been exacerbated by the nonfasted status of our patients. Centrally mediated bradycardia and hypotension may also explain the observed adverse events. Indeed, animal studies have shown that activation of the A_2_ receptors in the posterior hypothalamus leads to bradycardia and hypotension [30]. A direct activation of A_2_ receptors in the human brain has not been proved. It is possible that in susceptible individuals, a rapid increase in sympathetic tone mediated by direct activation of A_2A_ receptors in the sympathetic afferent nerves may result in reflex vagal discharge. This has already been proposed as a potential mechanism of action in adenosine-sensitive syncope [31].

### Lung disease

Although lung disease is not a contraindication to the use of regadenoson, we were initially cautious and used dobutamine in patients with persistent asthma or moderate to severe COPD. None of the patients with airways disease who underwent regadenoson stress developed wheeze, and hence clinically significant bronchoconstriction following regadenoson seems unlikely. Therefore, regadenoson is a safe option in patients with lung disease and this has been confirmed in a recent study [32].

The proportions of abnormal (62 %) and ischaemic (54 %) scans among our patients were significantly higher than those reported in a recent study by Rozanski et al. looking at temporal trends in the frequency of abnormal and ischaemic MPS over the last two decades in patients referred to Cedars-Sinai Medical Center [33]. According to the study, there has been a progressive decline in the prevalence of abnormal MPS studies from approximately 41 % in 1991 to only 9 % in 2009. Likewise, there has been a reduction in the prevalence of ischaemic scans from 30 % in 1999 to only 5 % in 2009. It is important to bear in mind that for the purposes of the study, 24 % of patients were excluded because of previous MI, mechanical revascularization, history of cardiomyopathy or valve disease. In contrast, our study included all patients referred for MPS regardless of medical history. Patients with known CAD, including those with previous MI or revascularization, comprised over half of our population. Our patients were on average 10 years older, with more cardiovascular risk factors and a higher frequency of anginal symptoms than in the Cedars-Sinai study, and this may partly explain the higher number of abnormal and ischaemic scans. These differences might also explain discrepancies found between this and recent studies on the side effect and safety profile of regadenoson, especially regarding the frequency of hypotensive episodes. In relative terms, our patient population was at “higher risk” of any complications during stress and hence likely to have exhibited a less favourable safety profile. Nonetheless, our findings indicate that regadenoson, as well as other forms of stress, are safe in this patient population with side effects and tolerability comparable to those observed in “lower risk” patients.

### Limitations

This study was observational and did not control for sample size. Although statistical analysis was limited by the large difference between group sizes, descriptive comparison was possible. As mentioned above, it was not possible to explain the mechanism of action by which regadenoson might cause vasovagal episodes. This observation is hypothesis-generating and therefore further research is needed to elucidate the underlying mechanism(s).

### Conclusion

In the largest European cohort to date, regadenoson combined with submaximal exercise has proved to be safe and well tolerated, notably also in patients with asthma and COPD. Most adverse events following regadenoson injection were vasovagal episodes. There is no clear direct mechanism by which regadenoson might cause vasovagal syncope.
